# Efficacy of PI monotherapy versus triple therapy for 1964 patients in 10 randomised trials

**DOI:** 10.7448/IAS.17.4.19788

**Published:** 2014-11-02

**Authors:** Jose Arribas, Pierre-Marie Girard, Nicholas Paton, Alan Winston, Anne-Genevieve Marcelin, Daniel Elbirt, Andrew Hill, Maria Blanca Hadacek

**Affiliations:** 1 IdiPAZ, Hospital la Paz, Madrid, Spain; 2 Maladies Infectieuses et Tropicales, Hopital Saint-Antoine, Paris, France; 3 Clinical Trials Unit, Medical Research Council, London, UK; 4 Jefferies Research Wing, St Mary's Hospital, London, UK; 5 Virology, Hopital Pitie Salpetriere, Paris, France; 6 AIDS Centre, Kaplan Medical Centre, Rehovot, Israel; 7 Pharmacology and Therapeutics, University of Liverpool, Liverpool, UK; 8 EMEA, Janssen, Issy-les-Moulineux, France

## Abstract

**Introduction:**

The efficacy of protease inhibitor monotherapy has been analyzed with different endpoints: initial HIV-1 RNA rebound, long-term HIV-1 RNA suppression after re-intensification, treatment-emergent drug resistance, neurocognitive testing and HIV-1 RNA in the cerebrospinal fluid (CSF).

**Methods:**

A PUBMED search identified nine randomised trials of PI monotherapy versus triple therapy in patients with HIV RNA<50 copies/mL at baseline. Results from the recently completed PROTEA trial were also included. The percentage of patients with HIV RNA suppression <50 copies/mL was analyzed using switch equals failure and intensification included endpoints (ITT). The number of patients with new drug resistance mutations, HIV RNA in the CSF or change in neurocognitive function was analyzed by treatment arm.

**Results:**

Four trials evaluated darunavir/r monotherapy (n=785: MONET, MONOI, MONARCH, PROTEA), five evaluated lopinavir/r monotherapy (n=592: OK-04, KalMo, KALESOLO, KRETA, MOST) and one evaluated both (MRC PIVOT, n=587). HIV-1 RNA suppression rates tended to be lower on PI monotherapy than triple therapy in “switch equals failure” analysis (76% vs 82%), but not when the outcome of intensification was included (87% vs 85%). There were small numbers of patients taking PI monotherapy with detectable HIV-1 RNA in the CSF, in three trials: PROTEA (n=2), MONOI (n=2) and MOST (n=5), but only two cases of CSF viral escape (MONOI). In two trials, there was no difference in neurocognitive test results between PI monotherapy and triple therapy, based on z-scores from five domains (in PROTEA, mean change in NPZ-5 score=0.0 for DRV/r monotherapy vs −0.10 for triple therapy); similar results were observed in the MRC PIVOT trial. The risk of treatment emergent NRTI or PI resistance (Table 1) was 11/973 (1.1%) for patients on PI monotherapy, versus 7/991 (0.7%) for patients on triple therapy.

**Conclusions:**

In 10 randomised trials of 1964 patients with HIV-1 RNA suppression at baseline, PI monotherapy showed a higher risk of HIV RNA elevations, and small numbers with HIV RNA detectable in CSF and concomitantly in the plasma. However there was no increased risk of treatment-emergent drug resistance, neurocognitive endpoints were not different between the arms and HIV-1 RNA suppression rates after intensification were similar between PI monotherapy and triple therapy.

**Table 1:** Number of patients with treatment emergent NRTI, NNRTI or PI resistance mutations

	NRTI or PI Resistance mutations	
	
Trial (n, duration)	PI/r	Triple therapy
**LPV/r trials**		
OK-04 (n=200, 96 wks)	2/100	2/98
Kalmo (n=60, 96 wks)	0/30	0/30
Kalesolo (n=186, 48 wks)	0/87	0/99
KRETA (n=88, 48 wks)	1/44	0/44
MOST (n=60, 24 wks)	0/29	0/31
DREAM (n=197, 96 wks)	3/11	
**DRV/r trials**		
MONET (n=256, 144 wks)	1/127	1/129
MONOI (n=246, 96 wks)	0/112	0/113
Monarch (n=30, 48 wks)	0/15	0/15
PROTEA (n=273, 48 wks)	0/137	0/136
**Mixed PI trials**		
PIVOT (n=587, 5 years)	7/291	4/296

